# Wired Differently? Brain Temporal Complexity and Intelligence in Autism Spectrum Disorder

**DOI:** 10.3390/brainsci15080796

**Published:** 2025-07-26

**Authors:** Moses O. Sokunbi, Oumayma Soula, Bertha Ochieng, Roger T. Staff

**Affiliations:** 1 Faculty of Health and Life Sciences, De Montfort University, Leicester LE1 9BH, UK; bertha.ochieng@dmu.ac.uk; 2 Faculty of Medicine of Sfax, University of Sfax, Sfax 3029, Tunisia; soula_oumayma@medecinesfax.org; 3 Department of Imaging Physics, Aberdeen Royal Infirmary, NHS Grampian, Aberdeen AB25 2ZD, UK; roger.staff@nhs.scot

**Keywords:** autism spectrum disorder, cognitive function, fuzzy approximate entropy, fuzzy sample entropy, Hurst exponent, intelligence, neural complexity, neurodevelopment, resting-state

## Abstract

**Background:** Autism spectrum disorder (ASD) is characterised by atypical behavioural and cognitive diversity, yet the neural underpinnings linking brain activity and individual presentations remain poorly understood. In this study, we investigated the relationship between resting-state functional magnetic resonance imaging (fMRI) signal complexity and intelligence (full-scale intelligence quotient (FIQ); verbal intelligence quotient (VIQ); and performance intelligence quotient (PIQ)) in male adults with ASD (*n* = 14) and matched neurotypical controls (*n* = 15). **Methods:** We used three complexity-based metrics: Hurst exponent (H), fuzzy approximate entropy (fApEn), and fuzzy sample entropy (fSampEn) to characterise resting-state fMRI signal dynamics, and correlated these measures with standardised intelligence scores. **Results:** Using a whole-brain measure, ASD participants showed significant negative correlations between PIQ and both fApEn and fSampEn, suggesting that increased neural irregularity may relate to reduced cognitive–perceptual performance in autistic individuals. No significant associations between entropy (fApEn and fSampEn) and PIQ were found in the control group. Group differences in brain–behaviour associations were confirmed through formal interaction testing using Fisher’s *r*-to-*z* transformation, which showed significantly stronger correlations in the ASD group. Complementary regression analyses with interaction terms further demonstrated that the entropy (fApEn and fSampEn) and PIQ relationship was significantly moderated by group, reinforcing evidence for autism-specific neural mechanisms underlying cognitive function. **Conclusions:** These findings provide insight into how cognitive functions in autism may not only reflect deficits but also an alternative neural strategy, suggesting that distinct temporal patterns may be associated with intelligence in ASD. These preliminary findings could inform clinical practice and influence health and social care policies, particularly in autism diagnosis and personalised support planning.

## 1. Introduction

Autism spectrum disorder (ASD) is a neurodevelopmental condition marked by impairments in social communication and interaction, along with restricted and repetitive behaviours [1]. ASD is also highly heterogeneous, manifesting with wide-ranging variability in symptom severity, cognitive abilities, neurodevelopmental trajectories, and adaptive functioning [2,3]. This heterogeneity poses a significant obstacle in identifying reliable biomarkers and neural signatures that can generalise across the spectrum. As a result, probing the subtle alterations in neural dynamics in ASD that may underlie diverse behavioural and cognitive presentations is a challenge in autism research.

Recent developments in brain signal analysis have introduced complexity science as a promising metric to interrogate neural variability and organisation. Brain signal complexity refers to the degree of irregularity, unpredictability, adaptability, and information richness in neural time series, providing a window into the system’s functional capacity, robustness, and flexibility [4]. Measures such as the Hurst exponent (H) and entropy (fuzzy approximate entropy (fApEn) and fuzzy sample entropy (fSampEn)) have been applied to resting-state functional magnetic resonance imaging (rs-fMRI) data to quantify long-range temporal correlations and signal irregularity across time [5,6,7]. These metrics, grounded in nonlinear dynamics, offer sensitivity to alterations in brain function that may remain undetected in conventional linear or region-specific fMRI analyses.

Brain signal complexity has been successfully employed in the study of various neuropsychiatric and neurodevelopmental disorders. For instance, decreases in complexity have been observed in individuals with attention-deficit/hyperactivity disorder (ADHD) [8], Alzheimer’s disease [9] and depression [10], suggesting reduced adaptability or neural inflexibility. Conversely, conditions such as schizophrenia have shown increased complexity, potentially reflecting unstable or dysregulated neural dynamics [5]. These findings collectively suggest that the directionality of complexity changes may vary across clinical populations, indicating that a disease-specific theoretical model is needed to explain these differences.

A key concept that informs our understanding of complexity in biological systems is the attractor model proposed by Vaillancourt and Newell [11]. The model proposes that the change in output complexity of a physiological system with age or disease depends on whether a fixed-point attractor or an oscillatory attractor governs the system. A fixed-point attractor refers to a state to which the system returns following perturbation, characterised by stability and reduced variability. In such systems, disease or ageing is typically associated with a loss of complexity, as seen in ADHD [8]. In contrast, oscillatory attractor systems may display increased complexity in pathological states, as fluctuations become more erratic or desynchronised. This principle was demonstrated in schizophrenics [5], where entropy measures revealed greater complexity in schizophrenic individuals compared to healthy controls.

This attractor-based model offers a compelling approach for examining complexity in ASD. Autism is not a singular entity but rather a spectrum of developmental trajectories influenced by genetic, neurobiological, and environmental factors [2]. Prior research has reported both increased and decreased brain complexity in autistic individuals, depending on brain region, developmental stage, and methodological approach [12]. These mixed findings may reflect the coexistence of multiple attractor dynamics within the autistic brain, possibly shaped by compensatory neural mechanisms or developmental adaptations. Notably, adults with ASD who are cognitively high-functioning may display neurotypical or even enhanced performance on specific cognitive tasks, potentially obscuring underlying differences in brain dynamics [13]. Furthermore, while studies have compared group-level differences in brain complexity between ASD and controls, fewer have investigated how these complexity measures relate to intelligence. Intelligence is known to correlate with efficient and flexible brain network dynamics in neurotypical populations, yet its relationship with complexity in autism remains underexplored. Understanding this association is crucial, as intelligence profiles in ASD often diverge from typical patterns, featuring discrepancies between verbal and non-verbal IQ or exceptional performance on tasks involving rule-based reasoning [13].

In this study, we investigate the relationship between brain signal complexity, as measured by the Hurst exponent (H), fuzzy approximate entropy (fApEn), and fuzzy sample entropy (fSampEn), and intelligence scores in adults with ASD compared to matched neurotypical controls. Whole-brain (global) metrics were computed by aggregating voxel-wise resting-state fMRI data across the entire brain. These voxel-level computations were used to derive global summary statistics for each subject. Specifically, we aimed to determine whether the direction and strength of the relationships between complexity and intelligence differ between groups, providing an insight into the neurocognitive architecture of autism.

Here, we hypothesise that the ASD brain will demonstrate significant differences from a non-ASD sample in whole-brain complexity metrics. We also hypothesised that any associations between complexity and intelligence would differ between groups, reflecting divergent neural strategies for supporting cognitive function.

By applying the dual-attractor theoretical approach to both whole-brain and regional analyses, our study aims to move beyond simple group comparisons and uncover deeper, functionally indicative patterns of brain signal complexity in ASD. Such insights may advance the development of individualised biomarkers and models that better reflect the heterogeneity of autism.

## 2. Materials and Methods

### 2.1. Participants

The present study utilised data from the Autism Brain Imaging Data Exchange (ABIDE I) database, specifically the University of Leuven: Sample 1 cohort. The final dataset comprised 29 participants aged 18 to 32 years, including 14 individuals with autism spectrum disorder (ASD) (mean age, 23.27 ± 2.92 years) and 15 age-matched typical controls (TC) (mean age, 21.86 ± 4.11 years). Participants diagnosed with ASD were drawn from a clinical sample at the Expertise Centrum Autism (ECA), Leuven University Hospital. Diagnosis was established according to DSM-IV-TR criteria by a multidisciplinary team. Inclusion criteria required a formal DSM-IV-TR diagnosis of autistic disorder and a raw score exceeding 60 on the Social Responsiveness Scale (SRS), Adult Version, as reported by a parent or guardian. Control participants were matched to the ASD group based on age, sex, and estimated IQ measures (full-scale IQ, verbal IQ, and performance IQ). They were screened to ensure the absence of neurological or psychiatric disorders, which were confirmed through lab-developed questionnaires and unstructured clinical interviews. ASD participants were recruited from previous studies at KU Leuven, while controls were recruited from the local community. All participants provided written informed consent. The study protocol was approved by the Ethics Committee for Biomedical Research at the Katholieke Universiteit Leuven, adhering to the Declaration of Helsinki.

### 2.2. Intelligence Measures

Estimates of intelligence quotient (IQ) indices: full-scale IQ (FIQ), verbal IQ (VIQ), and performance IQ (PIQ) were obtained from the ABIDE I dataset (University of Leuven, Sample 1 cohort). These estimates were derived using the Ward 7-subtest short form of the Wechsler Adult Intelligence Scale—III, as described in the ABIDE data documentation and supporting literature [14,15,16]. This short-form version offers a reliable approximation of the full WAIS-III battery and has been validated for clinical and research applications in both neurotypical and clinical populations [14,17]. These measures are designed to capture both general and domain-specific aspects of cognitive functioning and are particularly relevant in autism spectrum disorder (ASD), where dissociations between verbal and non-verbal abilities are common [18,19].

#### 2.2.1. Full-Scale Intelligence Quotient (FIQ)

The full-scale intelligence quotient (FIQ) is a global composite score that reflects an individual’s overall level of intellectual functioning. It is derived by combining scores from both verbal and performance subtests, providing a single summary index of cognitive ability. In this study, FIQ served as a general indicator of intelligence and was used to assess broad associations with brain complexity measures. FIQ is a significant predictor of educational and occupational outcomes and is often used as a benchmark for evaluating cognitive function in both typical and clinical populations [20].

#### 2.2.2. Verbal Intelligence Quotient (VIQ)

The verbal intelligence quotient (VIQ) represents the individual’s verbal comprehension, reasoning, and knowledge acquired from the environment. It is computed from verbal subtests, including Vocabulary, Similarities, Arithmetic, and Information. VIQ is considered a measure of crystallised intelligence and is sensitive to language development and educational exposure. In autism research, VIQ is particularly relevant due to the variability in verbal abilities among individuals on the spectrum, with some demonstrating strong verbal reasoning and others presenting with significant language impairments [21].

#### 2.2.3. Performance Intelligence Quotient (PIQ)

The performance intelligence quotient (PIQ) assesses non-verbal reasoning, visual-spatial processing, and fluid intelligence. It is derived from performance-based subtests such as Block Design, Matrix Reasoning, and Picture Completion. These tasks require perceptual organisation, visual-motor coordination, and problem-solving abilities, independent of verbal mediation. PIQ is particularly relevant in ASD populations, who often display relative strengths in non-verbal tasks despite verbal deficits, leading to characteristic discrepancies between VIQ and PIQ [22,23].

The inclusion of these three IQ indices enables a nuanced examination of how different facets of intelligence interact with brain signal complexity in autism and typical development. Furthermore, matching ASD and control participants on FIQ, VIQ, and PIQ helped to control for general cognitive ability as a confounding variable in group comparisons.

### 2.3. Brain Imaging

Prior to scanning, participants were familiarised with the MRI environment using a mock scanner and received both verbal and written instructions to minimise anxiety and motion during the actual scan. All imaging was conducted on a 3.0 Tesla Philips MRI scanner (Best, The Netherlands) equipped with an 8-channel phased-array head coil. The scanning protocol began with the acquisition of a high-resolution T1-weighted anatomical image, followed by a resting-state functional MRI (rs-fMRI) scan. During the resting-state scan, participants were instructed to lie still with their eyes open, fixate on a centrally presented white crosshair against a black background, and let their thoughts flow naturally without focusing on anything in particular. The rs-fMRI data were acquired using an echo-planar imaging (EPI) sequence with the following parameters: repetition time (TR) = 2000 ms, echo time (TE) = 30 ms, flip angle = 90°, field of view (FOV) = 224 mm, matrix size = 64 × 64, voxel size = 3.5 × 3.5 × 3.5 mm^3^, and number of slices = 37, covering the whole brain. A total of 250 volumes were collected throughout the resting-state scan. This setup ensured optimal spatial and temporal resolution for subsequent analysis of intrinsic brain activity.

### 2.4. Image Pre-Processing

Image pre-processing was carried out using the FMRIB Software Library (FSL; version 6.0.5.1), developed by the Oxford Centre for Functional MRI of the Brain (FMRIB), University of Oxford, Oxford, United Kingdom [24]. All procedures were executed in a containerised and reproducible cloud-based environment via the Neurodesk platform in Google Colab [25].

Each participant’s functional MRI dataset consisted of 250 volumes; the first 5 were discarded to eliminate scanner equilibration transients, yielding 245 volumes for analysis. Motion correction was performed using MCFLIRT (Motion Correction using FMRIB’s Linear Image Registration Tool), which realigns images using six degrees of freedom. Functional images were then co-registered to each subject’s T1-weighted anatomical scan, acquired using a Philips 3.0T MRI scanner (Best, The Netherlands) with a high-resolution MPRAGE (Magnetization-Prepared Rapid Gradient Echo) sequence with 1 mm isotropic voxels. The anatomical scans were pre-processed with FMRIB’s BET (Brain Extraction Tool) for skull stripping and FAST (FMRIB’s Automated Segmentation Tool) for bias correction and tissue segmentation.

Spatial normalisation of functional data to the standard Montreal Neurological Institute (MNI) space was completed using FLIRT (FMRIB’s Linear Image Registration Tool). Nuisance regression was performed to remove six motion parameters obtained from MCFLIRT. Subsequently, band-pass filtering (0.008–0.1 Hz) was applied to retain frequencies characteristic of resting-state neural activity while minimising physiological and scanner-related noise.

This sequence of nuisance regression followed by temporal filtering was selected to minimise spectral distortion in the signal, which is critical for accurate estimation of entropy-based complexity metrics. While more advanced denoising methods (e.g., ICA-AROMA, scrubbing) were not used due to the small sample size and risk of data loss, the current approach is consistent with standard practice in exploratory studies of resting-state temporal dynamics.

### 2.5. Complexity Metrics

#### 2.5.1. Hurst Exponent

The Hurst exponent (H) is a statistical measure used to assess the long-range temporal dependence and fractal properties of a time series [26]. In the context of fMRI analysis, it quantifies the degree to which BOLD signal fluctuations exhibit persistent, anti-persistent, or random behaviour over time. The Hurst exponent (H) takes values between 0 and 1, and it characterises the temporal correlation structure of a time series. Based on the value of H, a time series can be classified into three categories: (1) H = 0.5 represents a completely random process (white noise); (2) values between 0 and 0.5 indicate an anti-correlated (rough) time series; and (3) values between 0.5 and 1 suggest a positively correlated time series. [5]. In this study, the Hurst exponent was computed voxel-wise using dispersional analysis [27], a robust method that assesses how the variability (standard deviation) of the signal changes with increasing levels of temporal aggregation. This method is especially suitable for analysing biological signals, such as fMRI data, which may contain fractal and periodic components.

The core idea of dispersional analysis is to group the time series into non-overlapping windows of increasing size m, compute the standard deviation (*SD*) of these aggregated signals, and determine how *SD* changes as a function of m. Mathematically, this relationship follows a power law:

(1) $${SD}_{m}={SD}_{n}\cdot {\left(\frac{m}{n}\right)}^{H-1}\;H=\frac{\log\left({SD}_{m}/{SD}_{n}\right)\;}{\log\left(m/n\right)}$$

In Equation (1), *H* represents the Hurst exponent, *SD* denotes the variance or standard deviation, *m* indicates the element size used to compute *SD*, and *n* refers to the arbitrarily selected reference size, corresponding to the length over which the average is calculated [5]. In this study, *N* = 245 (the length of the fMRI time series), *m* = 2, and *n* = 1. A linear regression was performed on the log–log plot, and the resulting slope provided the best estimate of *H*. These estimates were used to generate whole-brain H maps, computed in MATLAB version R2023b. Our approach for estimating both the mean whole-brain *H* values and the *H* maps followed the procedure described by Sokunbi et al. [5].

#### 2.5.2. Fuzzy Approximate Entropy (fApEn)

Fuzzy approximate entropy (fApEn) is a nonlinear measure used to quantify the complexity, irregularity, and unpredictability of time series data. It is a refinement of the approximate entropy (ApEn) method proposed originally by Pincus [28]. It was designed to overcome limitations such as sensitivity to short data lengths and poor relative consistency. fApEn integrates fuzzy logic, which allows for a gradual and smooth definition of similarity between vectors rather than a binary classification, enhancing robustness against noise and data length variability. In traditional ApEn, the similarity between vectors is determined using a discontinuous Heaviside function, which can result in a loss of information in cases where data points are nearly, but not exactly, similar. fApEn replaces this with a continuous fuzzy membership function, introducing a smoother evaluation of similarity between embedded vectors in the time series.

The fuzzy membership function, derived from the concept of fuzzy sets introduced by Lotfi Zadeh [29], is employed in fApEn to provide a fuzzy measure of similarity between vectors based on their shapes. By replacing the hard thresholding of the Heaviside function with the smoother transition of the fuzzy membership function, the distinction between points becomes less rigid, allowing them to appear closer and more similar [30].

The calculation of the fApEn of a time series of length N involves the following steps [6]:

$$fApEn(m,r,N)=\Phi^{m}(r)-\Phi^{m+1}(r)$$

where

(2) $$\Phi^{m}(r)={\left[N-(m-1)\tau \right]}^{-1}\sum_{i=1}^{N-(m-1)\tau }\ln\left[C_{i}^{m}\left(r\right)\right]$$

and

(3) $$C_{i}^{m}\left(r\right)=\frac{1}{N-(m-1)\tau }\cdot \sum_{j=1}^{N-(m-1)\tau }D_{ij}^{m}$$

In Equation (2), *N* represents the number of time points, *m* denotes the pattern length, and *τ* is the time delay. In Equation (3), $D_{ij}^{m}$, the similarity measure is defined using a fuzzy membership function, which employs an automatic mirrored quadratic function. In this function, the fuzzy width is automatically determined based on the parameter *r*. $D_{ij}^{m}$ is given as

$$D_{ij}^{m}=u\left(d_{ij}^{m},r\right)$$

where the distance $d_{ij}^{m}$ between $X_{i}^{m}$ and $X_{j}^{m}$ (m-dimensional pattern vectors) is defined as

$$d_{ij}^{m}=d\left[X_{i}^{m},X_{j}^{m}\right]=\underset{k\varepsilon \left(0,m-1\right)}{\max}\left|u\left(i+k\right)-u0\left(i\right)-\left(u\left(j+k\right)-u0\left(j\right)\right)\right|$$

and

$$X_{i}^{m}=\left\{u\left(i\right),u\left(i+1\right),\ldots ,u\left(i+m-1\right)\right\}-u0\left(i\right)\;\left(i=1,2,\ldots ,N-(m-1)\tau \right)$$

Here, $u0\left(i\right)$ is a baseline value

$$u0\left(i\right)=\frac{1}{m}\sum_{j=0}^{m-1}u\left(i+j\right)$$

The symbol *r* represents a predetermined tolerance value, which is defined as

$$r=k\cdot std\left(T\right)$$

where *k* is a constant $\left(k>0\right)$, and $std\left(.\right)$ represents the standard deviation of the signal. The *degree* of similarity between the two patterns *i* and *j* of m measurements of the signal is determined by the fuzzy membership function, $u\left(d_{ij}^{m},r\right)$, a function of the distance between any pair of corresponding measurements of $X_{i}^{m}$ and $X_{j}^{m}$, with respect to the tolerance parameter *r*.

The fuzzy membership function applied in this study is constructed using a pair of mirrored quadratic curves, forming a sigmoid-like shape [6]. The basic equation is expressed below as a function of *x*, where *x* = distance/*r*:

$$\mu_{quadratic}\left(x\right)=\left\{\begin{array}{c} 0\leq x\leq 1:\frac{1}{2}(2-x^{2})\\ 1<x\leq 2:\frac{1}{2}{\left(2-x\right)}^{2} \end{array}\right.$$

An additional (optional) feature was incorporated to enable automatic adjustment of the fuzzy width based on the parameter *r*, as proposed by Xiong et al. [31].

Whole-brain fApEn maps were computed voxel-wise from the pre-processed resting-state fMRI data using custom MATLAB and C code. A time series length of 245 volumes (after discarding the initial 5) was used. The embedding dimension used was *m* = 2, the similarity criterion (threshold) was *r* = 0.25 × SD, and the time delay *τ* = 1 was also used. Voxels outside the brain were excluded by applying a threshold at 10% of the maximum signal intensity. This method allows for the generation of high-resolution spatial maps of signal complexity, providing insight into the temporal dynamics of brain activity.

#### 2.5.3. Fuzzy Sample Entropy (fSampEn)

Fuzzy sample entropy (fSampEn) is a refined metric for quantifying the complexity and irregularity of time series data, especially useful for physiological signals such as fMRI. It is based on sample entropy (SampEn), which was introduced as an improvement over approximate entropy (ApEn) by eliminating self-matching of template vectors and reducing bias associated with short datasets [32].

Unlike SampEn, which uses a binary similarity function (based on a fixed threshold), fSampEn incorporates fuzzy logic to evaluate the degree of similarity between sequences. This continuous similarity measure increases robustness to noise, improves monotonicity, and reduces dependence on data length.

Let {u(1),u(2),…,u(N)} be a time series of length *N*.

Form a set of *m*-dimensional vectors,

$$X_{i}^{m}=\left\{u\left(i\right),u\left(i+1\right),\ldots ,u\left(i+m-1\right)\right\},\;\;i=1,2,\ldots ,N-m$$

And for m+1,

$$X_{i}^{m+1}=\{u(i),u(i+1),\ldots ,u(i+m)\}$$

Compute the distance between two vectors $X_{i}^{m}$ and $X_{j}^{m}$ using the Chebyshev distance:

$$d_{ij}^{m}=\underset{0\leq k<m}{\max}|u(i+k)-u(j+k)|$$

Similarity is defined by a fuzzy function *μ*(*x*), where ${x=d}_{ij}^{m}/r$, and *r* is a similarity criterion (threshold) (*r* × standard deviation of the signal). We used the same fuzzy membership function used in fApEn above:

$$\mu_{quadratic}\left(x\right)=\left\{\begin{array}{c} 0\leq x\leq 1:\frac{1}{2}(2-x^{2})\\ 1<x\leq 2:\frac{1}{2}{\left(2-x\right)}^{2} \end{array}\right.$$

For each i, calculate

(4) $$\phi_{i}^{m}\left(r\right)=\frac{1}{N-m-1}\sum_{\begin{array}{c} j=1\\ j\neq 1 \end{array}}^{N-m}\mu \left(\frac{d_{ij}^{m}}{r}\right)$$

Then, average over all i to get

(5) $$\phi^{m}\left(r\right)=\frac{1}{N-m}\sum_{i=1}^{N-m}\phi_{i}^{m}\left(r\right)$$

Similarly, compute $\phi^{m+1}\left(r\right)$ using vectors of length m+1.

Finally, fuzzy sample entropy is defined as

$$fSampEn(m,r,N)=-ln\left(\frac{\phi^{m+1}\left(r\right)}{\phi^{m}\left(r\right)}\right)$$

Similar to the procedure used for fApEn, whole-brain fSampEn maps were computed on a voxel-wise basis from the pre-processed resting-state fMRI data. This was accomplished using a custom implementation in MATLAB and C programming environments. The time series length used was 245 volumes of fMRI data. The embedding dimension used was *m* = 2, the similarity criterion (threshold) was *r* = 0.25 × SD, and the time delay *τ* = 1 was also used. Voxels outside the brain were excluded by applying a threshold at 10% of the maximum signal intensity. Additionally, this method allows for the generation of high-resolution spatial maps of signal complexity, providing insight into the temporal dynamics of brain activity.

### 2.6. Statistical Analysis

Statistical analyses were performed using SPSS 28.0.1.1 (IBM Corp., Armonk, NY, USA). Mean group differences in the measured parameters, intelligence and whole-brain complexity, were tested using an independent *t*-test. Correlations between the complexity measures and intelligence scores (FIQ, VIQ, PIQ) were performed using a Pearson approach. Comparison between the correlations between the two groups was explored using Fisher’s *r*-to-*z* transformation using a two-tailed test. To formally assess whether the association between entropy and PIQ differed by group, we conducted regression analyses including interaction terms. Entropy metrics (fApEn and fSampEn) were mean-centred across the full sample, and the group variable (ASD = 1, Control = 2) was dummy coded. Interaction terms [Group × Centred Entropy (fApEn and fSampEn)] were computed and entered into linear regression models predicting PIQ. A significant interaction term would indicate a moderation effect, i.e., a group-specific difference in the entropy–PIQ association.

## 3. Results

The analysis found no significant differences between the group means for each parameter (Table 1). To control for multiple comparisons (21 total), False Discovery Rate (FDR) correction was applied (*q* = 0.05). Significant correlations after FDR correction were observed between the measured parameters, as shown in Table 2. As expected, there were correlations among the intelligence measures, as well as among the whole-brain complexity metrics.

**Figure 1:**
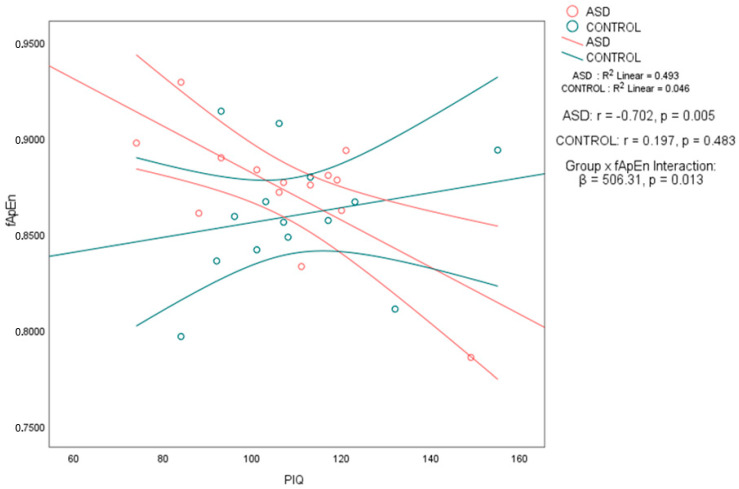
Overlay of the correlation between fuzzy approximate entropy (fApEn) and performance IQ (PIQ) in autistic and control participants. Scatterplots display the relationship between fApEn and PIQ for the autism spectrum disorder (ASD) group (*n* = 14) and the typically developing control group (*n* = 15). In the ASD group, there was a significant negative association between fApEn and PIQ (*r* = −0.702, *p* = 0.005), whereas the control group showed a non-significant positive association (*r* = 0.197, *p* = 0.483). Each point represents an individual participant. Linear regression lines with 95% confidence intervals are shown for both groups. A significant group × fApEn interaction was observed (*β* = 506.31, *p* = 0.013), indicating that the association between temporal brain complexity and PIQ significantly differed between groups.

**Figure 2:**
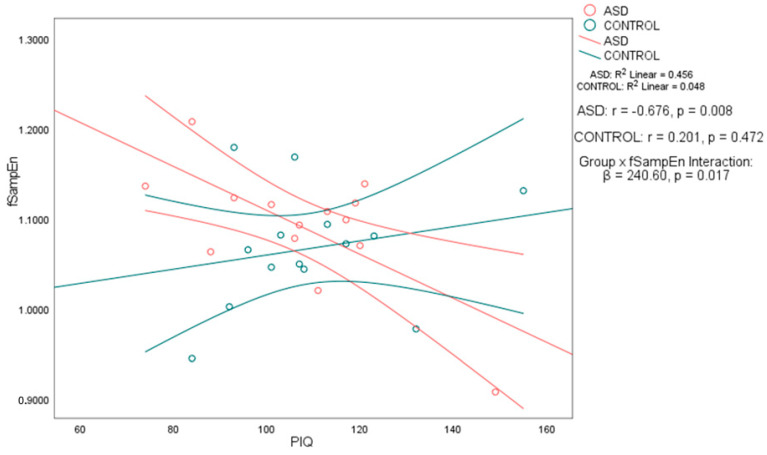
Overlay of the correlation between fuzzy sample entropy (fSampEn) and performance IQ (PIQ) in autistic and control participants. Scatterplots display the relationship between fSampEn and PIQ for the autism spectrum disorder (ASD) group (*n* = 14) and the typically developing control group (*n* = 15). In the ASD group, there was a significant negative association between fSampEn and PIQ (*r* = −0.676, *p* = 0.008), whereas the control group showed a non-significant positive association (*r* = 0.201, *p* = 0.472). Each point represents an individual participant. Linear regression lines with 95% confidence intervals are shown for both groups. A significant group × fSampEn interaction was observed (*β* = 240.60, *p* = 0.017), indicating that the association between temporal brain complexity and PIQ significantly differed between groups.

Notably, in the ASD group, performance IQ (PIQ) was significantly negatively correlated with both fApEn (*r* = −0.702, 95% CI [−0.898, −0.273], *p* < 0.01) and fSampEn (*r* = −0.676, 95% CI [−0.888, −0.227], *p* < 0.01). In contrast, the control group showed no significant associations between PIQ and either entropy metric, with weak positive correlations and wide confidence intervals that included zero: PIQ–fApEn (*r* = 0.197, 95% CI [−0.351, 0.644]) and PIQ–fSampEn (*r* = 0.201, 95% CI [−0.347, 0.647]) (see Table 3). To formally assess whether these associations differed by group, we applied Fisher’s *r*-to-*z* transformation. The PIQ–fApEn correlation was significantly stronger in the ASD group (*z* = −2.56, *p* = 0.0105), as was the PIQ–fSampEn correlation (*z* = −2.51, *p* = 0.012). These results support a group-specific relationship between performance IQ and whole-brain entropy, as shown in Table 3. No significant correlations were found between any of the intelligence measures and age, nor between the complexity measures and age. The scatter plots in Figure 1 and Figure 2 illustrate the association between PIQ and both fApEn and fSampEn, respectively. In the ASD group, the plots show that higher PIQ scores are associated with lower complexity (entropy) values, whereas the control group shows no clear trend, with data points appearing randomly distributed.

To further investigate whether the strength of the entropy–PIQ association differed significantly between groups, we conducted regression analyses with interaction terms. For fApEn, the interaction term was statistically significant (*β* = 506.31, *p* = 0.013), confirming that the relationship between fApEn and PIQ differs by group. A similar result was found for fSampEn (*β* = 240.60, *p* = 0.017) as shown in Table 4. These findings confirm a significant moderation effect of group on the entropy–PIQ association and validate the group-specific nature of the correlations previously reported.

## 4. Discussion

In this study, we examined whole-brain complexity metrics, including the Hurst exponent (H), fuzzy approximate entropy (fApEn), and fuzzy sample entropy (fSampEn), to explore their relationship with intelligence in individuals with ASD compared to age-matched neurotypical controls. While no significant differences were observed between groups in overall brain complexity measures, a notable finding emerged within the ASD group, where higher levels of entropy (as indicated by fApEn and fSampEn) were significantly correlated with lower performance IQ (PIQ) scores. A comparison of the correlation with the control group found a difference in the correlation between all three complexity measures. This inverse relationship suggests that increased neural complexity may be related to less efficient cognitive–perceptual processing in individuals with ASD.

These findings align with previous research indicating altered brain entropy in children with ASD [12]. Here, we report a whole-brain association in young adults. Other studies have reported increased entropy in specific brain regions of children with ASD, suggesting atypical neural dynamics [33]. Additionally, research has shown a shift toward randomness in ECG signals among adults with ASD, reflecting changes in the temporal structure of neural activity [34].

We hypothesised a whole-brain difference in entropy between the two groups, which we did not find. It is possible that we lacked the statistical power to detect such differences, or that our whole-brain approach is crude, averaging over the brain could be averaging out positive and negative regional differences that drive ASD presentation when compared to controls. Our findings support our second hypothesis, with significant associations with intelligence in individuals with ASD. The negative nature of the correlation indicates that those with ASD individuals with lower IQs have a more complex neuronal mechanism than those with ASD and higher IQs and controls with similar cognitive abilities. The last point must be qualified by the fact that the correlation in the control group was not significantly different from zero. That is, there are substantial parts of the brain, sufficient to affect the whole brain measure, operating under oscillatory attractor conditions that produce more complex signals when cognitive abilities are low. This complexity-intelligence relationship may reflect divergent neural strategies for supporting cognitive function, particularly in individuals with lower cognitive abilities. Higher brain entropy has been associated with greater cognitive flexibility [35] and intelligence in neurotypical populations. The inverse relationship observed in our ASD sample suggests a divergence in how neural complexity relates to cognitive function in this population. These findings provide further insight into how cognitive functions in autism may not only reflect deficits but also an alternative neural strategy for supporting complex tasks, which might differ from typical developmental trajectories [36]. These results align with the existing literature on neurodivergence in autism and the role of altered brain complexity and connectivity in shaping intelligence [37].

This study employed multiple complexity metrics, including the Hurst exponent (H), fuzzy approximate entropy (fApEn), and fuzzy sample entropy (fSampEn), to investigate the neural correlates of intelligence in ASD, providing a novel perspective on functional brain dynamics. However, the absence of significant group-level differences must be interpreted with caution and understood in the context of autism’s inherent clinical and neurobiological heterogeneity. Autism is not a monolithic condition; rather, it encompasses a wide range of presentations across cognitive ability, symptom severity, developmental history, and neural adaptation [3]. Complexity measures like entropy or fractal dimension often assume homogeneity in brain signal organisation, potentially obscuring group effects in ASD, where such uniformity rarely exists [38]. Although complexity metrics such as Hurst exponent, fApEn, and fSampEn have been successfully applied in neuroimaging studies [39,40], they are not without limitations. Previous studies have noted their sensitivity to pre-processing choices, scan length, and noise levels [41,42]. In the present study, we mitigated these issues through consistent pre-processing, the use of 245-volume time series, and parameter settings based on prior literature. While we acknowledge these methodological challenges, our findings remain robust and interpretable in the context of adult ASD fMRI analysis. Other models such as multiscale entropy, predictive coding, and information dynamics frameworks are also well-suited to resting-state fMRI data.

Recent studies employing graph-theoretical and multiscale network models [35,43] have provided valuable insights into the network-level correlates of intelligence. While these frameworks focus on inter-regional connectivity patterns, our study emphasises intra-regional temporal complexity. These approaches are complementary, and future work could benefit from integrating both to provide a more comprehensive understanding of brain–behaviour relationships in ASD.

The small sample size (14 autistic and 15 control participants) further limits statistical power. To address these limitations, future work should incorporate larger and more diverse samples and extend the analysis to include a regional assessment in addition to global measures. Incorporating multimodal imaging, developmental stratification, and behavioural subtyping will also be critical in future efforts to disentangle the nuanced relationship between brain complexity and cognition in ASD.

Our study highlights the nuanced relationship between brain complexity and intelligence in ASD. The observed association between increased entropy and lower PIQ in individuals with ASD suggests that atypical neural dynamics may impact cognitive–perceptual abilities. Although there are limitations due to the small sample size, these preliminary findings indicate that future research involving a larger group to elucidate the mechanisms underlying these relationships could inform and influence health and social care policies. This could lead to improved outcomes for individuals with ASD, ensuring that health and social care providers are well-equipped to offer effective support and resources for individuals with ASD and their families. Future studies should address current limitations, including small sample size, cross-sectional design, and limited behavioural characterisation by employing larger, developmentally stratified cohorts and incorporating/integrating machine learning or deep learning models capable of capturing nonlinear, temporal, and individualised patterns in brain data [44,45]. Ultimately, incorporating complexity-based metrics with advanced computational tools may enhance our understanding of autistic cognition and contribute to more personalised diagnostic and therapeutic strategies.

## 5. Conclusions

This study investigated the relationship between brain signal complexity and intelligence in individuals with autism spectrum disorder (ASD) and neurotypical controls using resting-state fMRI and three complexity-based metrics: Hurst exponent (H), fuzzy approximate entropy (fApEn), and fuzzy sample entropy (fSampEn). While no significant group-level differences in global brain complexity were observed, within the ASD group, whole-brain fApEn and fSampEn were significantly negatively correlated with performance IQ (PIQ).

Formal interaction testing using Fisher’s *r*-to-*z* transformation confirmed that these associations were significantly stronger in the ASD group than in controls, supporting a group-specific relationship between neural complexity and cognitive function. Complementing these findings, regression analyses including interaction terms further revealed that the association between entropy and PIQ was significantly moderated by group, strengthening the evidence for distinct brain–behaviour dynamics in autism.

The absence of overall group differences may reflect the heterogeneity of autism, potential compensatory mechanisms in high-functioning individuals, or the limitations of global metrics in capturing more subtle or spatially localised neurofunctional alterations. These findings underscore the importance of precision neuroscience approaches that account for individual variability and move beyond group-level comparisons.

Future research should address current limitations, including small sample size, cross-sectional design, and limited behavioural phenotyping. Longitudinal studies with larger, developmentally stratified cohorts, combined with machine learning and deep learning techniques, may better capture nonlinear, dynamic, and individualised brain–behaviour relationships. Integrating complexity-based neural markers with advanced computational models holds promise for advancing our understanding of autistic cognition and enabling more personalised diagnostic and therapeutic strategies.

## Figures and Tables

**Table 1:** Participants’ characteristics, mean intelligence scores and mean whole-brain complexity metrics.

	ASD		Control	
	Mean	SD	Mean	SD
Age	21.86	4.11	23.27	2.91
FIQ	109.43	13.09	114.80	12.86
VIQ	110.43	12.45	117.47	9.86
PIQ	107.36	18.88	109.13	17.68
Hurst Exponent	0.45	0.03	0.46	0.04
fApEn	0.87	0.03	0.86	0.03
fSampEn	1.09	0.07	1.07	0.07

**Table 2:** Pearson correlations between the intelligence measures (FIQ, VIQ and PIQ) and whole-brain complexity indices (H, fApEn and fSampEn) for the ASD sample (upper right, dark grey) and the control sample (lower left, light grey). ** indicates where the correlation differs from zero, *p* < 0.01. † Indicates where the correlation strengths differ between the ASD and the control group, *p* < 0.05.

	FIQ	VIQ	PIQ	Hurst Exponent	fApEn	fSampEn
FIQ		0.695 **	0.778 **	−0.114	−0.450	−0.419
VIQ	0.766 **		0.095	0.264	0.043	0.056
PIQ	0.899 **	0.419		−0.360	−0.702 **	−0.676 **
Hurst Exponent	0.428	0.249	0.418 †		0.014	−0.072
fApEn	−0.076	−0.411	0.197 †	−0.081		0.993 **
fSampEn	−0.060	−0.394	0.201 †	−0.105	0.991 **	

**Table 3:** Correlations between intelligence measures and whole-brain entropy metrics for ASD and control groups, with group comparisons.

Correlation	Group	*r*	95% CI	*z* (Fisher *r*-to-*z*)	*p*-Value (Group Difference)
PIQ–fApEn	ASD	−0.702	[−0.898, −0.273]		
	Control	0.197	[−0.351, 0.644]	−2.56	0.0105 *
PIQ–fSampEn	ASD	−0.676	[−0.888, −0.227]		
	Control	0.201	[−0.347, 0.647]	−2.51	0.012 *

Notes: * = significant group difference (*p* < 0.05, Fisher *r*-to-*z*); CI = confidence interval (95%)**.** Only PIQ correlations with entropy metrics showed significant group differences.

**Table 4:** Regression models testing Group × Entropy interaction predicting PIQ.

Predictor	B	Standard Error (SE)	*β*	*t*	*p*-Value	95% CI for B [Lower, Upper]
Model 1: fApEn						
Constant	109.52	9.76		11.23	<0.001	[89.42, 129.61]
Group	0.08	6.10	0.002	0.01	0.990	[−12.49, 12.65]
fApEn (centred)	−908.22	301.68	−1.67	−3.01	0.006	[−1529.55, −286.89]
Group × fApEn	506.31	188.24	1.49	2.69	0.013	[118.63, 893.99]
Model 2: fSampEn						
Constant	108.87	9.85		11.06	<0.001	[88.59, 129.15]
Group	0.39	6.16	0.011	0.06	0.950	[−12.30, 13.08]
fSampEn (centred)	−427.31	148.71	−1.59	−2.87	0.008	[−733.57, −121.04]
Group × fSampEn	240.60	93.94	1.41	2.56	0.017	[47.12, 434.08]

## Data Availability

The original contributions presented in this study are included in the article. Further inquiries can be directed to the corresponding author.
